# Avian Cytogenomics: Small Chromosomes, Long Evolutionary History

**DOI:** 10.3390/genes16091001

**Published:** 2025-08-25

**Authors:** Darren K. Griffin, Rafael Kretschmer, Denis M. Larkin, Kornsorn Srikulnath, Worapong Singchat, Valeriy G. Narushin, Rebecca E. O’Connor, Michael N. Romanov

**Affiliations:** 1 School of Natural Sciences, University of Kent, Canterbury CT2 7NJ, UK; 2 Animal Genomics and Bioresource Research Unit (AGB Research Unit), Faculty of Science, Kasetsart University, Chatuchak, Bangkok 10900, Thailand; kornsorn.s@ku.ac.th (K.S.); worapong.singc@ku.ac.th (W.S.); 3 Departamento de Genética, Instituto de Biociências, Universidade Federal do Rio Grande do Sul, Porto Alegre 91540-000, RS, Brazil; rafael.kretschmer@ufrgs.br; 4 Royal Veterinary College, University of London, London NW1 0TU, UK; dlarkin@rvc.ac.uk; 5 Vita-Market Lab, 69035 Zaporizhya, Ukraine; valnarushin@gmail.com; 6 L. K. Ernst Federal Research Center for Animal Husbandry, Dubrovitsy, Podolsk Municipal District, Moscow Oblast 142132, Russia

**Keywords:** birds (Aves), cytogenomics, avian genomes, genome organization, chromosomes, chromosomal rearrangements, genome evolution, molecular genetic approaches, genomic technologies, adaptation

## Abstract

This review considers fundamental issues related to the genomics of birds (Aves), including the special organization and evolution of their chromosomes. In particular, we address the capabilities of molecular genetic/genomic approaches to clarify aspects of their evolutionary history, including how they have adapted to multiple habitats. We contemplate general genomic organization, including the small size and typical number of micro/macrochromosomes. We discuss recent genome sequencing efforts and how this relates to cytogenomic studies. We consider the emergence of this unique organization ~245 million years ago, examining examples where the “norm” is not followed. We address the functional role of synteny disruptions, centromere repositioning, repetitive elements, evolutionary breakpoints, synteny blocks and the role of the unique ZW sex chromosome system. By analyzing the cytogenetic maps and chromosomal rearrangements of eight species, the possibility of successfully applying modern genomic methods/technologies to identify general and specific features of genomic organization and an in-depth understanding of the fundamental patterns of the evolution of avian genomes are demonstrated. An interpretation of the observed genomic “variadicity” and specific chromosomal rearrangements is subsequently proposed. We also present a mathematical assessment of cross-species bacterial artificial chromosome (BAC) hybridization during genomic mapping in the white-throated sparrow, a species considered a key model of avian behavior. Building on model species (e.g., chicken), avian cytogenomics now encompasses hundreds of genomes across nearly all families, revealing remarkable genomic conservation with many dynamic aspects. Combining classical cytogenetics, high-throughput sequencing and emerging technologies provides increasingly detailed insights into the structure, function and evolutionary organization of these remarkable genomes.

## 1. Introduction

Birds constitute the phylogenetic class Aves—warm-blooded tetrapods with unique phenotypic features, enormous biodiversity and particular challenges associated with studying the organization, function and evolution of their genomes [1]. This genome is typically relatively small and conservative with low karyotypic inter-species variability and a predominance of microchromosomes [2,3,4].

Over the past century, a vast amount of information has been accumulated on the karyology, genetics, physiology, biochemistry and genome evolution of the chicken (*Gallus gallus*) [5,6,7,8] followed by that of various other avian species [3,9,10,11]. The chicken genome project [12,13,14,15,16] generated genomic resources that have been, and will continue to be, used for comparative purposes, ultimately to elucidate fundamental evolutionary processes among birds in general [17,18,19]. Farm and domestic avian species are studied especially because of the economic importance of components of their disease ecology and zoonotic transmission as well as phenotypic traits associated with growth, reproduction and development [20].

Aves are a large and diverse class of vertebrates, with 10,500–11,000 species, about 2000 genera, 250 families and 40 orders [21] that display remarkable adaptations to flight, migration and survival in a variety of terrestrial and aquatic habitats. They inhabit every continent as well as remote oceanic islands, including the harsh climates of the Arctic and Antarctic, high mountains and hot deserts. Bird species are divided into two major groups (or infraclasses), Palaeognathae (ratites and tinamou) and Neognathae (flying, or secondarily flightless, birds), the differences between which are based on features of the morphology of the palatine formation and have been confirmed at the molecular level using DNA–DNA hybridization and genome sequencing [9,10,11,22].

The generation of the first available avian genome sequence for the domestic chicken [12,13] served as a starting point for the subsequent generation of genomic maps and sequences of the turkey and zebra finch [23,24,25,26]. Subsequently, 48 more avian genomes were sequenced [27], which, along with advancements in modern high-throughput molecular genetic methods and genomic technologies, enabled a significant breakthrough in large-scale fundamental avian research. Thus, new, in-depth information was obtained on their macroevolution and phylogenetics [9,28,29]. Recently, Stiller et al. [11] analyzed the genomes of 363 bird species, representing 218 taxonomic families and covering 92% of all avian families. By focusing on intergenic regions and employing coalescent-based methods, the authors reconstructed a well-supported phylogenetic tree.

The interest in fundamental research on birds continues unabated, including in areas such as the study of general and specific aspects of the avian genome’s structure, function and evolution [30]. In particular, one key point of work in this area is the elucidation of chromosomal rearrangements, both intrachromosomal and interchromosomal, which enables us to envision specific paths of avian genome evolution [29,31]. The purpose of this review is to give a brief description of the genomic organization as it relates to the genome organization and chromosomes of birds and to demonstrate the analysis of evolutionary changes (rearrangements in chromosomes) using the example of a few species, the genomes of which are among both the most and the least studied to date.

## 2. General Organization of Bird Genomes

The avian genome is characterized by a relatively small and conservative size of 1–2 Gb. Basic karyotyping usually results in a total diploid number of chromosomes, 2*n* = 70–82, most of which are tiny microchromosomes measuring 0.3–3 microns [32] that do not change substantially from species to species (with rare exceptions). The karyotype of the chicken, a classic model for many fields of biology [5], has a diploid chromosome number of 2*n* = 78 and, except for the fusion of chromosome 4 to a large microchromosome, probably represents a genome organization very close to the ancestral pattern. The haploid chicken genome contains about 1.2 × 10^9^ bp (or 1.2 Gb). This is 2.75 times less than that of mammals, corresponding, for instance, to only 39% of the size of the human genome [32].

Until about 2010, the chicken was the only bird with a sequenced genome, published in 2004 [12,13] and serving as a reference in genome browsers and databases [33]. Based on the chicken genome, a number of fundamental features of the organization of the genetic apparatus of birds have been described. It has been established that 10–20% of their genome is repeats, as opposed to 30–50% in mammals. Among the interspersed repeats, which comprise 9–11% of the chicken genome, there are no short interspersed nuclear element (SINE)-class blocks, a unique case among vertebrates. Satellite DNA occupies 5% of the chicken assembly [8]. At the same time, there are heavy isochores of the H4 type, not observed in other groups studied.

According to the International Chicken Genome Sequencing Consortium [12,13], microchromosomes comprise 18% of the female *Gallus gallus* genome and contain 31% of the species’ genes. In total, about 18,000 predicted genes have been identified in the chicken, compared with 23,000 in humans. The recombination rate is 6.4 cM/Mb in microchromosomes and 2.8 cM/Mb in macrochromosomes, compared to 1–2 cM/Mb in most mammals. Microchromosomes are enriched in CpG islands and heavy isochores and replicate early in the S phase. Three classes of telomeric repeats have been described in the chicken. The largest of these, up to 2 Mb, are found only in birds (e.g., [4,34,35]).

The spatial organization of avian macro- and microchromosomes has garnered significant attention in recent research. In birds, the presence of numerous microchromosomes contributes to a distinct three-dimensional genome architecture, with these small chromosomes typically occupying central nuclear regions and exhibiting high levels of transcriptional activity [36,37,38]. The spatial segregation of macro- and microchromosomes may reflect functional compartmentalization, potentially linked to the evolutionary stability of avian karyotypes [39,40]. Future studies integrating cytogenomics, 3D genome mapping and epigenomics will be essential for elucidating how spatial genome architecture influences chromosomal evolution and phenotypic diversification in birds.

Birds typically possess compact genomes with a lower content of repetitive sequences compared to mammals; nevertheless, repetitive elements have played a fundamental role in avian chromosomal evolution [41,42]. Long interspersed nuclear elements (LINEs), particularly CR1 elements, are the most prevalent transposable elements (TEs) in bird genomes and have been associated with chromosomal rearrangements, centromere repositioning and heterochromatin formation [12,13,30,41]. Microsatellite expansions, often lineage-specific, are enriched in pericentromeric and telomeric regions, particularly on the avian-specific W chromosome [43,44,45]. The accumulation of repetitive sequences on the W chromosome contributes to its accelerated differentiation and pronounced structural divergence [46,47,48]. Elucidating the distribution and activity of these repetitive elements is essential for understanding the evolutionary dynamics of both euchromatic and heterochromatic regions in avian chromosomes.

In addition to comparisons with mammals, avian chromosomes are compared to those of crocodilians, which are the closest extant relatives of birds and offer valuable context for understanding archosaur genome evolution. The availability of high-quality crocodilian genome assemblies through the International Crocodilian Genomes Working Group (ICGWG) enables comparative analyses of genome structure, repeat content and synteny. Unlike birds, crocodilians retain large chromosomes with fewer microchromosomes and show high genome stability, characterized by conserved synteny and low chromosomal rearrangement rates. Both groups exhibit relatively slow genome evolution [49]; however, avian genomes have undergone extensive compaction, microchromosome proliferation, increased recombination rates and reduced transposable element content—features likely linked to adaptations for powered flight and body size reduction [28]. These contrasting genomic landscapes reflect lineage-specific innovations that arose after the divergence from their common archosaur ancestor.

## 3. Sequencing Bird Genomes

Approximately six years after the publication of the chicken genome, data on the zebra finch (*Taeniopygia guttata*) and turkey (*Meleagris gallopavo*) began to appear [23,24,25,26]. Results of partial decoding of the genetic apparatus of such species as the collared flycatcher (*Ficedula albicollis*), large ground finch (*Geospiza magnirostris*), medium ground finch (*Geospiza fortis*), Puerto Rican amazon (*Amazona vittata*), rock pigeon (*Columba livia*) and others [50,51,52,53] were also obtained (see a list of initially sequenced bird genomes in Table 1). At the same time, effective methods for assembling new sequences of bird genomes using cytogenetic information were proposed [4,18,54].

Under the auspices of the Genome 10K project, the initial goal was to increase the number of sequenced bird genomes to 1000 [56,57]. However, plans were soon announced to sequence all extant bird species [58]. The Bird 10K project was initiated in 2015 by the Avian Phylogenomics Consortium, which planned to generate draft genome sequences within five years. The sequencing data and additional information on the morphological, physiological, ecological and behavioral characteristics of each bird species will be used for studies of avian evolution, ecology, population genetics, neurobiology, development and conservation. Such data may also be helpful for research into animal-to-human infections, such as avian influenza. Based on this information, it is becoming increasingly possible to construct a genomic tree of life for modern birds, as well as to identify the relationships between their genotypes and phenotypes, as well as their genetic, evolutionary and biogeographic relationships. Researchers have also planned to assess the biodiversity of birds and the influence of environmental factors (including anthropogenic ones) on their evolution [4]. In the first phase of this project, representatives of 34 bird orders were sequenced [27]. In the second phase, approximately 240 families are planned to be sequenced; in the third phase, 2250 genera; and in the fourth phase, the remaining 8000 species. To the best of our knowledge, the current number of bird species with sequenced genomes is 363 [11]; however, this is far too large a number to list in Table 1. Progress in sequencing, cloning of genomes and molecular cytogenetic techniques has created prerequisites for the subsequent development of comparative and evolutionary cytogenomics of birds [1,4,28,54,59,60,61] as outlined below.

## 4. Evolution of Birds and Their Genomes

**Figure 1:**
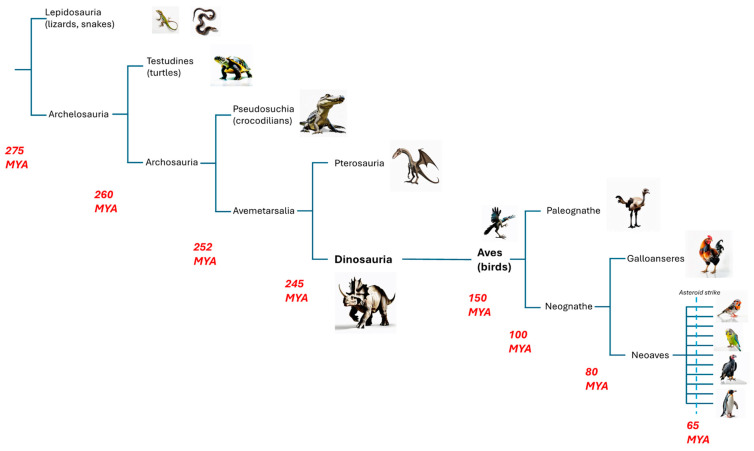
Phylogeny and divergence times of birds and their ancestors (adapted and modified from [3,64]). The time scale is given in million years ago (MYA).

Evolutionarily, birds are a monophyletic group of homeothermic (warm-blooded) tetrapods that share a distant common ancestor with humans and other mammals that lived approximately 310 million years ago (MYA). Synapsids (mammals and their extinct predecessors), testudines (turtles) and diapsids (other reptiles and birds) diverged between 310 and 350 MYA [20] (Figure 1).

Birds evolved from theropod dinosaurs approximately 150 MYA [62,63,64,65,66,67], with the earliest examples being *Vegavis* [68,69] and *Archaeopteryx* [70,71] from the Late Jurassic (~150 MYA) (Figure 1). Fossils of most modern bird orders appear in the early Cenozoic (<65 MYA). Comparisons of avian mitochondrial DNA with modern reptiles suggest that birds are most closely related to crocodilians, with the divergence between the two lineages estimated to have occurred 210–250 MYA [72].

In 2014, the Avian Genome Project examined the comparative and evolutionary aspects of the genome sequences of 48 species. The resultant data on bird evolution were reported in a special issue of *Science* [27,28] and a series of other publications that accompanied this issue (e.g., [29]). One of the main results of these studies is the assertion that, despite the high conservatism in this group of higher vertebrates, many non-neutral evolutionary changes are found in both coding genes and non-coding regions, correlating with the different life conditions of different bird species [4,27,28]. Using the six best-studied genomes, it was possible to reconstruct the putative ancestral karyotype of dinosaurs [29,59,67,73,74]. It was also possible to reconstruct the karyotype structure of the common ancestor of birds [29,61,73]. In addition, the existence of mechanisms for maintaining the karyotype and genome structure in the avian lineage was demonstrated [4,28].

The degree of evolutionary divergence between the genomes of closely and distantly related bird species can also be assessed using DNA hybridization with bacterial artificial chromosome (BAC) and overgo probes [25,26,75,76,77,78,79,80,81,82] (see also Section 5, Section 6, Section 8 and Section 9).

## 5. Bird Chromosomes and Their Evolution

The study of chromosomes in the infraclasses of ratites (Palaeognathae, formerly Ratitae) and neognath birds is primarily associated with the use of the fluorescence in situ hybridization (FISH) method, microchromosomal dyes and specific BAC clones [60,83,84,85,86,87,88], which made it possible to describe in more detail the microchromosomes that are poorly studied even in the chicken [4]. The study using BAC clones focused on chicken microchromosomes 10–28 [3]. Only recently have BAC probes been developed for chicken microchromosomes 29–38 [8]. However, these newly developed probes have not yet been applied in comparative cytogenetic studies across other avian species.

Using FISH, the presence of rarer interchromosomal rearrangements was demonstrated, e.g., in the ostrich and somewhat more frequent ones in the chicken lineage [29]. It is also worth noting the work that described a special microchromosomal pool of special dyes, which demonstrated a clear predominance of microchromosomal rearrangements over macrochromosomal ones in several studied bird species [31,89]. However, if we take most bird orders, then interchromosomal rearrangements involving microchromosomes are generally a rare phenomenon in birds [3,82]. Fissions of avian microchromosomes have not been documented to date, suggesting that these elements are generally resistant to chromosomal breakage. The only reported case of microchromosome disruption involves *Veniliornis spilogaster*, in which an ancestral microchromosome (homologous to GGA12) fused with a macrochromosome. Subsequently, an inversion occurred, with one of the breakpoints located in the middle of the region corresponding to GGA12 [90].

In studies of the karyotype of ratites (or running birds), in particular the ostrich and emu, minimal evolutionary variability of chromosomes or even its absence has been described [29,91,92]. The chromosomes of neognath birds can be examined using the example of studying the genome of the pigeon (*Columbia livia*) as well as representatives of the order Charadriiformes. The pigeon has a typical karyotype for birds (2*n* = 80). Zoo-FISH experiments (comparative hybridization between chromosomes of distant species) with DNA probes of Charadriiformes indicate the conservatism of large macrochromosomes (1–4). At the same time, studies on Charadriiformes highlight the origin of medium macrochromosomes as a result of tandem fusions of microchromosomes [93]. Birds of the order Falconiformes are characterized primarily by a reduction in the number of chromosomes, in particular, to 2*n* = 50 in the peregrine falcon (*Falco peregrinus*). This reduction is accompanied by tandem fusions of microchromosomes with macrochromosomes [80,94,95]. Chromosome studies in Charadriiformes are particularly compelling due to the remarkable karyotypic variation observed between species. This diverse order, which includes sandpipers, plovers, gulls and auks, exhibits a wide range of diploid numbers, from 2*n* = 42 in *Burhinus oedicnemus* [96] to 2*n* = 96 in *Scolopax rusticola* [81], highlighting the dynamic nature of chromosomal evolution in the group. Cytogenetic analyses have shown that this variability is primarily driven by extensive chromosomal fissions and fusions, involving both macrochromosomes and microchromosomes. In the wattled jacana (*Jacana jacana*), for instance, multiple chromosomal rearrangements among macrochromosomes have been documented, resulting in a stable diploid number of 2*n* = 82 [97]. Collectively, these findings emphasize the complexity of chromosomal evolution in Charadriiformes and underscore the importance of cytogenetic approaches for understanding avian genome architecture.

Recently, Duchêne et al. [98] discovered drivers of avian genomic change, suggesting that the majority of the rate variation happens along the phylogenetic tree’s more recent branches that are connected to modern bird families. Following the Cretaceous–Paleogene transition, microchromosomes underwent rapid changes, according to additional tests on axes of rate variation. Such evolutionary pulses appear to correlate with the ecological diversity represented in longer tarsuses and are consistent with significant changes in the genetic machinery for meiosis, heart function, RNA splicing, surveillance and translation. These combined studies [98] provide a complex picture of avian evolution, showing that throughout the early Paleogene, the progenitors of the most varied bird lineages experienced significant genomic changes associated with mutation, gene usage and niche expansion.

Germline-restricted chromosomes (GRCs) are additional chromosomes present exclusively in the germline and show striking similarities to supernumerary or B chromosomes, particularly in their dispensability and variable presence. While B chromosomes are frequently described as widespread across plants, animals and fungi, but notably absent in birds due to their reduced genome size, recent cytogenetic and genomic studies have challenged this notion. It is now evident that GRCs are likely present in all songbirds, indicating an ancient and conserved feature dating back approximately ~50 million years [99,100].

Despite their evolutionary conservation, GRCs display considerable variability in both size and gene content [99,101]. Genetically, they are highly dynamic and show extensive divergence from both autosomes and sex chromosomes [37,100,102]. In their composition, being repeat-rich and gene-poor, GRCs resemble the W chromosome [46,47], though they are even more variable and evolutionarily labile [103,104].

Given their unique features and potential roles in genome plasticity and germline–soma differentiation, the inclusion of a discussion on GRCs would strengthen this review and provide a more complete picture of avian chromosome evolution.

## 6. Genome Evolution: Synteny Disruptions, Centromere Repositioning and Repetitive Elements

Eukaryotes and their genomes appear to evolve through micro- and macrorearrangements [105,106,107]. Microrearrangements include inversions of paired genes and insertions and deletions of single genes, whereas macrorearrangements are large chromosomal rearrangements that are crucial for the evolution of genome structure and adaptability. In addition, sequencing data from several eukaryotes indicate that TEs and endogenous retroviruses have been shown to be sources of genetic innovation and serve regulatory functions in many organisms [30,108]. Comparative sequence analysis in mammals indicates that macrorearrangements are localized to telomere and centromere regions [109]. Studies describing the dynamics of mammalian genome evolution indicate the reuse of genomic regions for independent evolutionary breaks in different evolutionary lineages [110], as well as the presence of breaks that are more prone to rearrangements [111].

Centromere repositioning (CR) is a common phenomenon in eukaryotes [112]. It entails the inactivation of an existing centromere and the emergence of a new one along a chromosome. Following CR, the chromosome’s morphological markers stay in the same order, but the major constriction and centromeric function are localized to the new location. The architecture of chromosomes is significantly impacted by these processes, as shown in studies using locus-specific BAC and P1-derived artificial chromosome (PAC) clones in primates [111,113,114,115], equines [112], birds [85,86,116,117] and other organisms. According to these results, karyotype development in certain species may be significantly influenced by the CR phenomenon, which could have implications for speciation and population dynamics [20]. Although CR is considered an important event in mammalian chromosomal evolution, relatively little information is available on the organization of centromeres and CRs in birds. The DNA sequences of the centromeric regions are largely unknown and, therefore, represent gaps in the current assembly of chicken chromosome sequences. The small number of well-defined centromere-specific repeat units in birds includes the 41–42 bp tandem chicken nuclear-membrane-associated (CNM) repeat that is mainly localized in a set of microchromosomes, including the W sex chromosome [118]; the partially inverted repeat (PIR) found on chicken chromosome 8 [119]; and a few others. Within the draft chicken genome sequence, single CNM repeats sharing 95% identity were established only on chromosomes 23 and 28 (GGA23 and GGA28) and their centromeres were assigned to CNM positions accordingly. In addition, 53 CNM repeats were identified on unaligned contigs [119]. Repeat families play a dynamic function because these sequences are typically not conserved across species in the same order or even family and cannot be utilized for centromere localization or interspecies DNA hybridization. The so-called alphoid highly repetitive DNA sequences in the human genome are associated with marked linkage disequilibrium, indicating that changes in the organization of centromeric regions have led to selective sweeps [120].

FISH mapping of chicken BAC clones for chicken chromosome 4 (GGA4) on red grouse metaphases revealed that the order of the loci was the same in both species, but a neocentromere arose during divergence [116]. Similar neocentromere formation on Japanese quail chromosome 4 was detected by BAC-FISH mapping at the lampbrush stage of chicken and quail chromosomes [121]. The centromeres of chicken and quail chromosomes 4 apparently formed independently following centric fusion of the ancestral chromosome 4 and a microchromosome. Using immunostaining with antibodies against cohesin subunits, Krasikova et al. [122] showed that cohesin-enriched structures, similar to the so-called centromeric protein bodies, are characteristic of lampbrush chromosomes in Galliformes. Their centromeric location was confirmed by FISH experiments using specific DNA probes, including BAC clones. The fission predicted to be centromeric in the current GGA3 sequence assembly was found to correspond to a non-centromeric CNM repeat cluster on the q-arm of GGA3; the centromere itself is proposed to be placed at a different position. Thus, at least in Galliformes, the centromeres on GGA3 and GGA4 appear to be formed de novo during karyotype evolution in birds [20].

FISH hybridization of BAC probes thus allows the identification of organizational and structural changes in avian genomes that may point the way to further whole-genome sequencing studies. Comparative approaches to the study of genome sequences of birds, reptiles and other vertebrate classes contribute to the broad advancement of knowledge about comparative aspects of avian genome organization and to the elucidation of how genomic changes influence the evolutionary diversification and adaptive radiation of birds. The study of homologues of GGA3 and GGA4 in other species is an example of the information that can be obtained about these processes [20].

The evidence supports the important role of repetitive elements, such as retroposons, in the dynamic aspects of chromosomal evolution, including both micro- and macrorearrangement events. Crombach and Hogeweg [123] tested an evolutionary model in which genomes with retroposons and breakage-and-repair machinery are exposed to a changing environment. Retroposon-mediated rearrangements were shown to be a useful mutational operator for short-term adaptation to a new environment. Just being able to rearrange chromosomes, however, does not give one an edge over genomes that exclusively experience single-gene insertions and deletions. Instead, genome restructuring is required, since genes that need to be amplified (or deleted) in a new environment are often clustered, allowing rapid rearrangement-based adaptation to the environment. Crombach and Hogeweg [123] showed that genomes containing retroposons, starting from a random gene order, will eventually become organized, allowing for (rapid) rearrangement-based adaptation to the environment. In other words, this model may serve as a proof of principle that genomes can structure themselves to enhance the beneficial effects of chromosomal rearrangements [20].

Genome size in eukaryotes is mediated primarily by the amplification and attenuation of retroelement copies during evolution. The nearly 380-fold variation in genome sizes observed in modern vertebrates can be attributed to the repeat profiles of the genomes of the major amniote clades [124]. Efficient approaches to sequencing and identifying major repeat families in phylogenetically diverse avian taxa make it possible to characterize the repeat content and organization in pericentromeric regions and to assess whether centromeric regions are dynamic and provide contributions of noncoding DNA to the maintenance of synteny. It is desirable to significantly expand our understanding of the role of CRs in birds through the use of cell culture and the identification of informative hybridization probe sequences [26,125].

## 7. Evolutionary Breakpoints and Synteny Blocks in Avian Genomes

Bird genomes are known for their small size and conserved gene order. Many genome regions remain in the same arrangement across avian species, forming large homologous synteny blocks (HSBs). In contrast, evolutionary breakpoint regions (EBRs) mark the points where chromosomes have broken and rejoined in meiosis during evolution. Studying HSBs and EBRs helps us understand how bird genomes have changed over time and how genome structure contributes to both conserved and novel traits. Avian genomes have evolved more slowly compared to those of mammals. Karyotype and genome sequence studies (e.g., [27,28]) have found that modern avian genomes demonstrate strong conservation in gene order, chromosome number and architecture. Approximately 76% of the chicken genome is found in HSBs shared with other birds and reptiles. HSBs are not just ancestral sequence associations; they often contain genes critical for basic cellular and developmental functions. Farré et al. [126] found that genes within avian and archosaurian HSBs are significantly enriched for essential processes, including transcriptional regulation and embryonic development. For example, genes governing limb formation, neural tube development and morphogenesis are enriched in HSBs. Microchromosomes are composed mainly of long HSBs and are densely packed with functional genes. They have higher GC content and gene density and fewer repeats, all of which support their evolutionary stability and different evolutionary trajectory compared to macrochromosomes [127].

These findings align with mammalian studies. For instance, in humans and other mammals, conserved synteny blocks tend to overlap with developmental genes and topologically associating domains (TADs), which are three-dimensional genomic structures that co-localize genes with their regulatory elements [110,128,129]. The maintenance of TADs in birds could explain why HSBs are resistant to rearrangement.

EBRs are not random; they often occur in genomic regions enriched in repetitive elements, segmental duplications and open chromatin, making them hotspots for rearrangement. In birds, Farré et al. [126] demonstrated that EBRs are significantly associated with genes involved in neurogenesis and sensory perception, traits that vary widely among bird lineages. For example, rearrangements near neurodevelopmental genes in parrots and songbirds may underlie their advanced vocal learning capabilities. In other vertebrates, the functional roles of EBRs are well documented. Franke et al. [130] demonstrated that in mammals, EBRs frequently disrupt TAD boundaries, resulting in gene misregulation and the emergence of novel expression patterns. In primates, some EBRs brought enhancers into proximity with new genes, thereby affecting traits such as limb morphology. In marsupials, EBRs have been implicated in immune gene rearrangements, highlighting their role in adapting to ecological pressures [131]. EBRs in birds frequently coincide with bursts of species diversification or phenotypic innovation. Zhang et al. [28] noted a higher density of EBRs in birds with complex behaviors, like parrots and hummingbirds. In parrots, an EBR involves the rearrangement of genes related to the forebrain and motor control—traits essential for their unique vocal learning skills. Interestingly, songbirds also display elevated EBR rates compared to their sister non-songbirds. Rearrangement events affecting brain development genes may have facilitated the emergence of their specialized song circuits. Another example comes from the falcon lineage, which has undergone extensive chromosome reshuffling. Although falcons retain many of the same genes as other birds, their genomes show a dramatically different structure. This suggests that the rearrangements may contribute to lineage-specific adaptations in vision, flight dynamics or predatory behavior.

Together, EBRs and HSBs provide an example of how evolution acts on genome architecture. HSBs ensure the conservation of critical gene networks, protecting against harmful rearrangements that could disrupt essential functions. Meanwhile, EBRs enable genomic flexibility, allowing for the formation of new genes (resulting from duplications followed by diversification), deletions, interactions and regulatory rewiring. This dual strategy, stability through HSBs and innovation through EBRs, may help explain how birds diversified into over 10,000 species while retaining a compact, streamlined genome. It also emphasizes the functional balance between conservation and change that is characteristic of genome evolution in general.

## 8. Sex Chromosomes and Other Features

**Figure 2:**
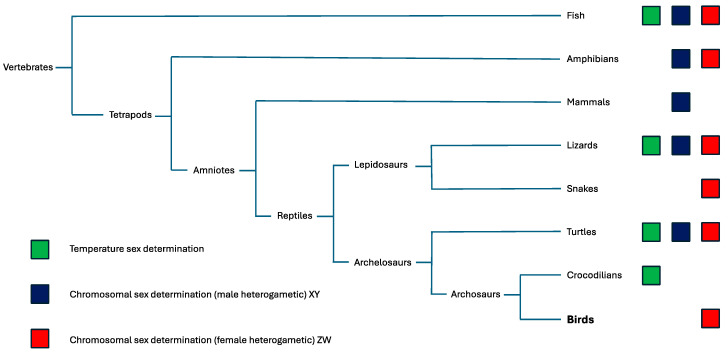
Vertebrate phylogeny and modes of sex determination in different taxa (adapted and modified from [132]). Red and black represent genetic sex determination with female and male heterogamety, respectively, whereas green corresponds to temperature-dependent sex determination. Birds and snakes have female heterogamety (albeit with sex chromosomes indistinguishable in the Palaeognathae), mammals have male heterogamety and other groups have different combinations.

Among vertebrates, temperature sex determination, male heterogamety (predominantly XY) and female heterogamety (predominantly ZW) are represented (Figure 2).

Birds are characterized by a special ZW genetic sex determination variant fundamentally different to that of mammals, with a Z-chromosomal sex-determining gene *DMRT1* and a heterogametic female sex [133]. Male birds are therefore predominantly ZZ and females predominantly ZW. The role of the W chromosome remains unclear; for example, its participation in the epigenetic regulation of the *DMRT1* gene activity is suggested [134]. It should be noted that in many bird species, primarily in the infraclass Palaeognathae, the sex chromosomes are virtually homomorphic, while heteromorphism and the appearance of a heteromorphic W chromosome are characteristic of the infraclass Neognathae [135]. The phenomenon of non-specific synapsis of sex chromosomes in meiosis in neognaths is also interesting [136]. The presence of sex-controlling genes in the W chromosome is suggested, for example, by the discovery of ZZW females in the Kentish plover (*Charadrius alexandrinus*) [137] and the suggestion of the presence of a gene F factor controlling the work of sex-determining Z-chromosomal genes [138]. The total number of W-chromosomal genes is estimated at 28, and the role of these genes remains controversial [48].

W-chromosomal probes can be used as markers of differentiation and heterochromatization of this microchromosome in birds, including ratites, for which there are currently only a few studies [139]. There are data on the genetics of the chicken W chromosome [48]. The problem of the evolutionary structure of this microchromosome, its genetic content and participation in sex determination remains open [4]. The length of the chicken W chromosome assembly reaches 14.2 Mb, in which repetitive sequences occupy 87% of the chromosome, including 4.9 Mb satellite DNA which is the most abundant repeat class [8].

The Z chromosome has undergone multiple inversions throughout avian evolution, resulting in diverse morphological variants across species. Despite these structural changes, its gene content remains largely conserved within specific syntenic blocks [140]. In contrast, the W chromosome has accumulated large amounts of repetitive DNA, including TEs and satellite DNAs (satDNAs), which contribute to its structural heterogeneity [30,43,45,46,47]. Consequently, the size of the W chromosome can vary considerably, even among closely related species [141].

In rare cases, avian sex chromosomes have been involved in chromosomal rearrangements with autosomes, resulting in the formation of multiple sex chromosome systems. Two such instances have been documented: one in a penguin species and another in the genus *Sula* [142,143]. In the Adélie penguin (*Pygoscelis adeliae*, Spheniscidae, Sphenisciformes), males possess a diploid number of 2*n* = 96 with a Z_1_Z_1_Z_2_Z_2_ sex chromosome system, whereas females have 2*n* = 95 with a Z_1_Z_2_W system [142]. Similarly, in the genus *Sula*, at least two species (*S*. *dactylatra* and *S*. *leucogaster*) exhibit sexual dimorphism in diploid numbers (2*n* = 76 in males and 2*n* = 75 in females), due to the presence of a multiple sex chromosome system of the Z_1_Z_1_Z_2_Z_2_/Z_1_Z_2_W type. Interestingly, while this sex chromosome configuration appears to be shared among *Sula* species, it has been described only in *P*. *adeliae* within penguins.

While the W chromosome in most neognaths is highly degenerated due to the accumulation of repetitive sequences, several paleognathous birds, such as ostriches and emus, retain relatively conserved and slowly evolving W chromosomes, likely reflecting reduced repetitive element activity or different chromosomal dynamics [46,144,145].

## 9. An Example of Analysis of Cytogenetic Maps and Chromosomal Rearrangements in Eight Birds

**Figure 3:**
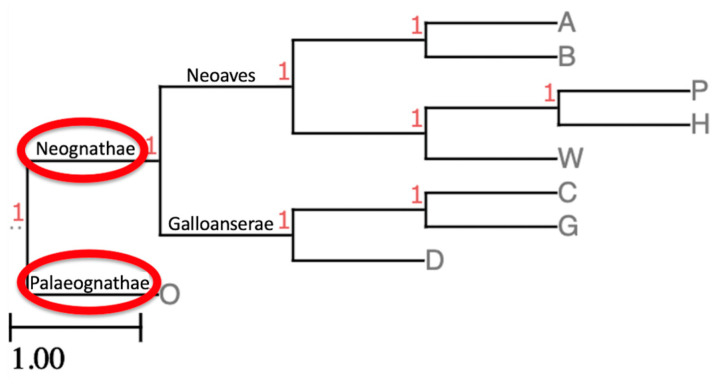
Phylogenetic tree for eight neognaths [17]: canary (A), blackbird (B), pigeon (P), houbara (H), woodcock (W), chicken (C), guineafowl (G) and duck (D). The hypothetical ancestor of Neognathae is circled in red. The ostrich (O), a representative of the ratites (Palaeognathae), is used as an outgroup. The cladogram is based on the avian phylogenetic tree [10] using the ETE Toolkit [146].

In this section, we consider a specific example of the study of the nature of chromosomal rearrangements in avian macrochromosomes, which was carried out at the University of Kent, Canterbury, UK [17,87]. For this purpose, the authors [17,87] selected eight species representing 6 of the 32 orders of neognaths: the common blackbird (*Turdus merula*) and Atlantic canary (*Serinus canaria*), both representatives of the order Passeriformes; the Eurasian woodcock (*Scolopax rusticola*; order Charadriiformes); the chicken (*Gallus gallus*) and guineafowl (*Numida meleagris*), both representatives of the order Galliformes; the African houbara (*Chlamydotis undulata*; order Bustardiformes); and the pigeon (*Columba livia*; order Columbiformes) and duck (*Anas platyrhynchos*; order Anseriformes). The position of these eight species on the phylogenetic tree of birds is conventionally presented in Figure 3.

A universal avian hybridization probe set, consisting of chicken BAC clones [80,147], was used to screen chromosomes of eight species using cross-species FISH. The probe set was designed to track the evolution of macrochromosomes by generating comparative cytogenetic maps. This approach enables the identification of chromosome rearrangements, such as fissions, fusions, duplications and inversions, all of which contribute to chromosomal changes that affect speciation and phylogenetic relationships between species. Based on the obtained comparative cytogenetic maps [87], the genome of a hypothetical ancestor of neognath birds was reconstructed using the MLGO program [148]. The analysis of observed chromosomal rearrangements in eight species relative to the ancestral genome was carried out visually and using the GRIMM program [149].

A summary of chromosomal rearrangements identified for the eight studied bird species is presented in Table 2 [17,87]. The smallest number of all rearrangements was characteristic of the chicken (4) and guineafowl (6), representatives of the order Galliformes, an earlier systematic group in the evolution of birds. The largest number of all chromosomal rearrangements (16) was found in the woodcock.

**Figure 4:**
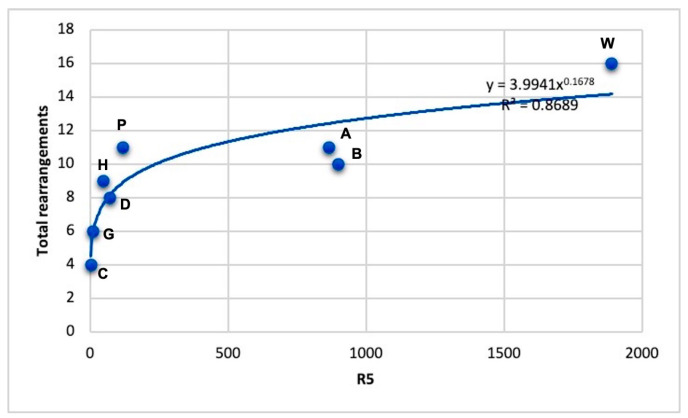
Graphical dependence (according to [17,87]) of the integral indicator of genomic “variadicity” and specific chromosomal rearrangements (*R*_5_; *x*-axis) on the total number of all rearrangements (*y*-axis) for eight Neognathae representatives: canary (A), blackbird (B), pigeon (P), houbara (H), woodcock (W), chicken (C), guineafowl (G) and duck (D).

For further analysis of chromosomal rearrangements, Romanov et al. [17] selected five traits (factors) that have individual values for each of the eight compared bird species: *R*_1_ is the success rate of cross-species FISH hybridization, *R*_2_ is the total number of all rearrangements, *R*_3_ is the number of all intrachromosomal rearrangements, *R*_4_ is the number of all interchromosomal rearrangements and *R*_5_ is the ratio of the species’ double set of chromosomes to the typical avian karyotype, taken as 80 chromosomes (2*n*/80). As can be seen from Table 2, each of these five factors has its own specific variability and nature (magnitude and spread) of values. For example, factor *R*_4_ for the duck, pigeon and bustard is 0, since interchromosomal rearrangements were not found in these species; if we take the double set of chromosomes (2*n*) used in calculating the *R*_5_ indicator, then here we have, as a rule, only two available values (78 and 80), and only the woodcock (96) stands out from this series, and so on in the case of other factors. In other words, it is not easy to integrally generalize such different-quality factors into any one indicator. Therefore, as a solution to this problem, Romanov et al. [17] proposed transforming the data for five factors, which have very different qualities, by ranking them. For example, instead of the available values for the number of all interchromosomal rearrangements, which are used as *R*_4_: 1, 2, 0, 0, 0, 1, 3, 8, appropriate ranks (from 1 to 5) to each value (in ascending order of the original values from 0 to 8) were assigned: 2, 3, 1, 1, 1, 2, 4, 5. The authors [17] performed similar transformations (rankings) for the remaining four factors. Then, the transformed values for the five factors were multiplied together for each species, which yielded a new integral indicator (*R*_5_). Next, a graph was constructed for eight species, where the total number of all rearrangements (*R*_2_) is plotted along the *y*-axis, and the integral indicator *R*_5_ is plotted along the *x*-axis (Figure 4). For the eight points obtained on the graph in Figure 4, a power function was then selected, which yielded a very high correlation (*R* = 0.9321) and which can be interpreted as an adequate dependence for reflecting the “variadicity” of the eight bird genomes relative to each other and in evolutionary terms as a whole. For clarity, although the term “variadicity” is used in mathematics and computer science to refer to the ability of a function to accept a variable number of arguments, in this case the authors [17] did not use it here in its strict meaning. Rather, they introduced it to interpret genomic variability within the class Aves.

Interestingly, in the graph of Figure 4, the authors [17] observed a close location of the chicken and guinea fowl (C, G), belonging to the order Galliformes, and the canary and blackbird (A, B) from the order Passeriformes, with a very distant location of the woodcock (W; order Charadriiformes), which is in good agreement with the phylogeny we adopted for these eight species (Figure 3). The pigeon (P), in principle, also occupies a very isolated position, and next to it is the houbara (H; Figure 4), which also fits into the accepted phylogeny (Figure 3). At the same time, the duck (D) is closer to these two species, moving away from the chicken and guinea fowl (Figure 2), with which the duck taxonomically forms a common clade—the superorder Galloanserae (Figure 3). The observed discrepancy in the distribution of the eight species in the graph of Figure 4 with the generally accepted phylogenetic concepts for birds can be explained, since the FISH method was used in this work, from which it is difficult to expect very high accuracy. Nevertheless, even with this method, the authors [17] were able to see some general patterns characteristic of the accepted taxonomy (phylogeny) and the evolution of the genomes of these species.

The proposed interpretation of genomic “variadicity” and specific chromosomal rearrangements (Figure 4) is also consistent with the information accumulated to date on the general organization and variability of the genomes of various avian species and taxonomic groups [28,29,31]. Thus, the chicken genome, which occupies the lowest position in the graph in Figure 4 (with minimal “variadicity” and rearrangement rates), is considered to be the closest to the ancestral genome of birds [29], while the genomes of passerines, as the most diverse and evolutionarily more recent order of birds, exhibit a very high rate of evolution [9,28]. Apparently, the genome of the woodcock from the order Charadriiformes is characterized by a very high degree of chromosomal changes, which is expressed both in an increased diploid set of chromosomes and in a large number of chromosomal rearrangements, as was demonstrated in this study [17,87] using the woodcock genome as an example.

## 10. An Example of Evaluation of Cross-Species BAC Hybridization in the White-Throated Sparrow Genome Mapping

**Figure 5:**
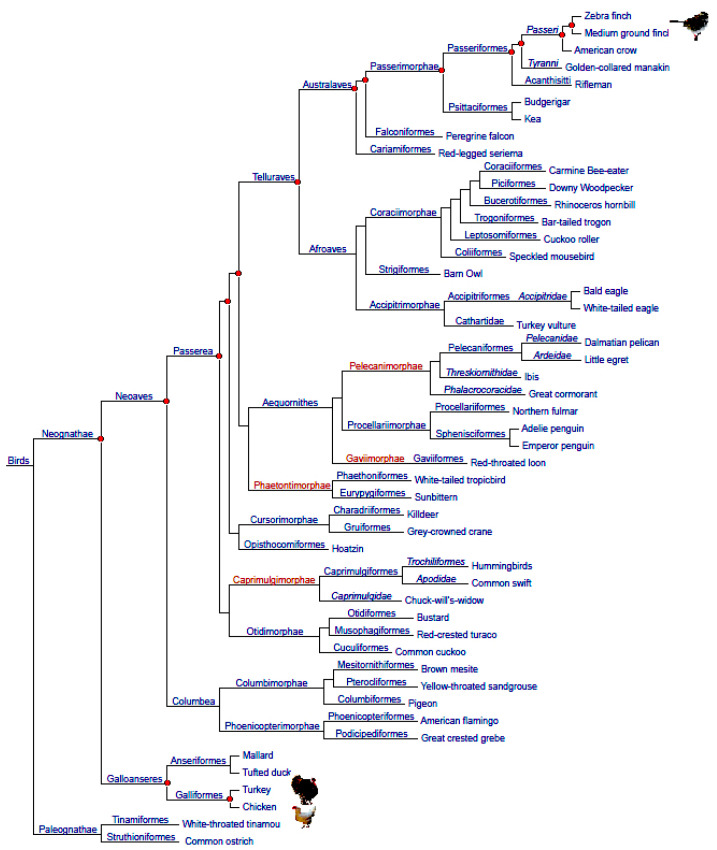
Phylogenetic tree of birds (according to [9,150,151]). The chicken and turkey are located near the bottom of the tree, on the basal branch of Neognathae, and passerines (Passeri) are at the very top of the tree, on the evolutionarily youngest branch. Evolutionary distances between different taxa are presented conditionally.

The white-throated sparrow (*Zonotrichia albicollis*) is a passerine bird species known for its morphological, behavioral and chromosomal variability, which represents a completely new model system for studying the genomic mechanisms underlying the variable behavioral repertoire, as well as aspects of population biology, reproduction and adaptation in this species. It has been previously shown that this sparrow’s variability can be a consequence of chromosomal rearrangements (inversions) on its chromosome 2 (ZAL2), which is characterized by heterogeneity in the form of two different plumage color morphs—brown (ZAL2/ZAL2) and white (ZAL2/ZAL2^m^) (see review in [78]). Romanov et al. [150] performed a mathematical evaluation of the results of cross-species DNA hybridization obtained earlier in the process of genomic mapping in the white-throated sparrow using the BAC library of this species [78]. To construct a comparative genomic map of ZAL2 and other chromosomes, Romanov et al. [78] used a genomic BAC clone library of the white-throated sparrow, CHORI-264, which was screened using overgo probes constructed based on DNA sequences of two representatives of the order Galliformes, the chicken (*Gallus gallus*) and the turkey (*Meleagris gallopavo*), as well as another representative of passerine birds, the zebra finch (*Taeniopygia guttata*). The position of the chicken, turkey and passerines on the phylogenetic tree of birds is shown in Figure 5. The obtained BAC library screening data [78] were used for further mathematical evaluation of interspecies DNA hybridization.

Using the cross-species DNA hybridization approach, Romanov et al. [78] screened the *Zonotrichia* library. They developed a first-generation comparative physical map of the species’ genome based on the BAC library against the chicken, turkey and zebra finch reference genomes. The map includes 640 *Zonotrichia* BAC clone alignments for 77 gene loci and provides a resource for further refinement of genomic regions and identification of candidate genes that may be subject to chromosomal rearrangements and contribute to the observed behavioral variation [78]. The results of the white-throated sparrow genomic BAC library screening and the relative success rates of the cross-species DNA hybridization are presented in Table 3 and Table 4.

As follows from Table 3, there is some variability in the values of the percentage of successful DNA hybridization and the number of positive BAC clones per DNA probe depending on the chromosome type. One possible explanation for the scattering of these parameters may be a different frequency of chromosomal rearrangements in the karyotypes of the studied species. At the same time, in the considered bird species, the double set of chromosomes (2*n*) was practically the same and equal to 78 in the chicken, 80 in the turkey and zebra finch and 82 in the white-throated sparrow. Therefore, the search for any mathematical correlations between the efficiency of DNA probes and their binding to a specific chromosome (or groups of chromosomes) would not be particularly meaningful. Thus, it can be assumed that the value of successful DNA hybridization can be influenced by factors that are more dependent on the conservatism of DNA regions (coding versus non-coding) used to construct the probes, as well as on the degree of evolutionary divergence of the compared bird species (Table 4).

**Figure 6:**
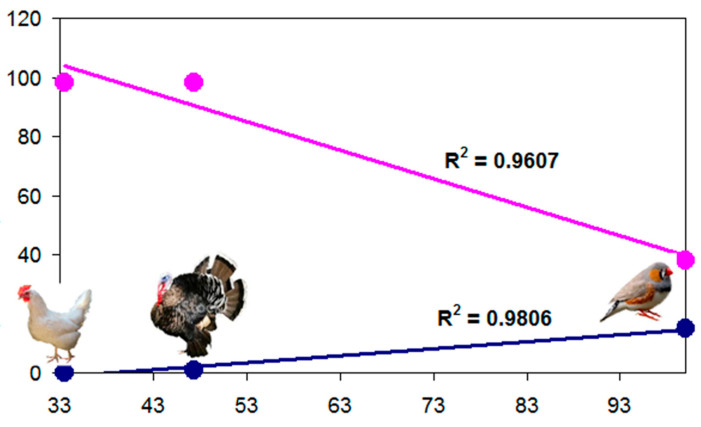
Correlation between the success rate of probes (%; *x*-axis) and the evolutionary divergence of birds (million years ago; *y*-axis) resulting from cross-species DNA hybridization (according to [78,150]). Dark blue points and trend line, number of nodes on the phylogenetic tree; pink points and trend line, divergence time.

Based on the data in Table 4, graphs were obtained for the correlation between the success rate of using probes and the evolutionary divergence of birds as a result of cross-species DNA hybridization (Figure 6). In this case, the correlation coefficients for the graphs showing the dependence of the success rate of probes on both the number of nodes on the phylogenetic tree (Figure 5) separating the white-throated sparrow from the chicken and turkey and on the magnitude of the evolutionary divergence between these birds, expressed in millions of years (Table 4), were high: *R* = 0.9903 and *R* = 0.9802, respectively.

Thus, the cross-species DNA hybridization data and the linear correlation graphs obtained on their basis (Figure 6) are adequate to reflect the nature of the evolutionary process for the selected species and systematic groups of birds. In further studies in this direction, the proposed approach to the mathematical assessment of cross-species DNA hybridization [150] may be useful in selecting bird species for comparison and characterization of their genomic evolution (e.g., [155,156,157]).

## 11. Conclusions

Over the past two decades, the field of avian genomics has advanced rapidly, building upon early work with model species like *Gallus gallus* to encompass hundreds of bird genomes across nearly all avian families. These efforts have revealed the remarkable genomic conservation of aspects of birds’ genomes, including their small genome sizes, predominance of microchromosomes and relatively low content of repetitive elements. At the same time, studies have uncovered many dynamic aspects of genome evolution, such as isolated chromosomal rearrangements, the role of TEs and structural differentiation of sex chromosomes. The combination of classical cytogenetics, high-throughput sequencing and emerging technologies such as 3D genome mapping is providing increasingly detailed insights into the structural, functional and evolutionary organization of bird genomes. Continued exploration of both well-studied and understudied species promises to refine our understanding of genome evolution in Aves and to shed light on the broader mechanisms shaping birds’ genomic diversity.

## Figures and Tables

**Table 1:** List of sequenced bird genomes in the NCBI database (as of 2021 [4]).

Species	Latin Name	Order	Chromosome No. in the Genome Assembly	Genome ID in the NCBI Database ^1^	Mitochondrial Genome
maguari stork	*Ciconia maguari*	Ciconiiformes	31	92,799	sequenced
California condor	*Gymnogyps californianus*	Accipitriformes/Cathartiformes	30	730	–
brown creeper	*Certhia americana*	Passeriformes	24	103,027	–
white wagtail	*Motacilla alba*	Passeriformes	31	43,097	sequenced
white-breasted antbird	*Rhegmatorhina hoffmannsi*	Passeriformes	35	92,741	–
great tit	*Parus major*	Passeriformes	31	12,863	sequenced
brown-headed cowbird	*Molothrus ater*	Passeriformes	34	88,920	–
western jackdaw	*Coloeus monedula*	Passeriformes	29	93,095	–
barn swallow	*Hirundo rustica*	Passeriformes	39	73,420	–
house sparrow	*Passer domesticus*	Passeriformes	30	17,653	sequenced
Swainson’s thrush	*Catharus ustulatus*	Passeriformes	43	86,530	sequenced
common yellowthroat	*Geothlypis trichas*	Passeriformes	34	86,337	–
European robin	*Erithacus rubecula*	Passeriformes	33	92,589	–
Sunda zebra finch	*Taeniopygia guttata*	Passeriformes	41	367	sequenced
Eurasian chaffinch	*Fringilla coelebs*	Passeriformes	30	34,546	–
small tree finch	*Camarhynchus parvulus*	Passeriformes	31	84,210	–
yellow-rumped warbler	*Setophaga coronata*	Passeriformes	31	46,404	sequenced
collared flycatcher	*Ficedula albicollis*	Passeriformes	30	11,872	sequenced
New Caledonian crow	*Corvus moneduloides*	Passeriformes	36	85,337	sequenced
white-rumped munia	*Lonchura striata*	Passeriformes	31	43,765	sequenced
lance-tailed manakin	*Chiroxiphia lanceolata*	Passeriformes	35	86,579	–
superb fairywren	*Malurus cyaneus*	Passeriformes	25	86,232	sequenced
garden warbler	*Sylvia borin*	Passeriformes	37	92,826	–
hooded crow	*Corvus cornix*	Passeriformes	29	18,230	sequenced
rifleman	*Acanthisitta chloris*	Passeriformes	38	32,002	sequenced
Eurasian blackcap	*Sylvia atricapilla*	Passeriformes	35	8421	–
black-throated flowerpiercer	*Diglossa brunneiventris*	Passeriformes	31	103,900	–
black-capped chickadee	*Poecile atricapillus*	Passeriformes	19	33,953	sequenced
European turtle dove	*Streptopelia turtur*	Columbiformes	33	81,804	–
rock dove	*Columba livia*	Columbiformes	29	10,719	sequenced
ruddy duck	*Oxyura jamaicensis*	Anseriformes	34	87,936	sequenced
African pygmy goose	*Nettapus auritus*	Anseriformes	34	87,938	sequenced
freckled duck	*Stictonetta naevosa*	Anseriformes	34	87,939	sequenced
mallard (domestic duck)	*Anas platyrhynchos*	Anseriformes	41	2793	sequenced
mute swan	*Cygnus olor*	Anseriformes	36	38,225	sequenced
Muscovy duck	*Cairina moschata*	Anseriformes	30	8552	–
tufted duck	*Aythya fuligula*	Anseriformes	36	33,654	sequenced
black-headed duck	*Heteronetta atricapilla*	Anseriformes	34	87,937	sequenced
northern flicker	*Colaptes auratus*	Piciformes	12	96,575	–
southern red-fronted tinkerbird	*Pogoniulus pusillus*	Piciformes	46	96,564	sequenced
downy woodpecker	*Picoides pubescens*	Piciformes	46	32,059	sequenced
grey crowned crane	*Balearica regulorum*	Gruiformes	37	17,144	sequenced
emu	*Dromaius novaehollandiae*	Casuariiformes	31	123	sequenced
red-legged seriema	*Cariama cristata*	Cariamiformes	52	31,967	sequenced
great potoo	*Nyctibius grandis*	Nyctibiiformes	38	92,333	sequenced
European nightjar	*Caprimulgus europaeus*	Nyctibiiformes	37	101,473	–
cuckoo	*Cuculus canorus*	Cuculiformes	41	32,170	sequenced
red junglefowl (chicken)	*Gallus gallus*	Galliformes	41	111	sequenced
turkey	*Meleagris gallopavo*	Galliformes	36	112	sequenced
Japanese quail	*Coturnix japonica*	Galliformes	29	113	sequenced
helmeted guineafowl	*Numida meleagris*	Galliformes	30	14,094	sequenced
budgerigar	*Melopsittacus undulatus*	Psittaciformes	32	10,765	sequenced
kākāpō	*Strigops habroptila*	Psittaciformes	25	75,115	sequenced
monk parakeet	*Myiopsitta monachus*	Psittaciformes	25	40,151	–
blue-fronted amazon	*Amazona aestiva*	Psittaciformes	29	40,915	–
Abyssinian ground hornbill	*Bucorvus abyssinicus*	Bucerotiformes	41	86,364	–
northern carmine bee-eater	*Merops nubicus*	Coraciiformes	36	31,978	sequenced
razorbill	*Alca torda*	Charadriiformes	26	84,534	sequenced
European golden plover	*Pluvialis apricaria*	Charadriiformes	38	100,067	sequenced
common tern	*Sterna hirundo*	Charadriiformes	27	66,333	sequenced
yellow-throated sandgrouse	*Pterocles gutturalis*	Pterocliformes	36	32,063	sequenced
gyrfalcon	*Falco rusticolus*	Falconiformes	24	43,830	sequenced
peregrine falcon	*Falco peregrinus*	Falconiformes	19	132	sequenced
lesser kestrel	*Falco naumanni*	Falconiformes	27	44,448	sequenced
Anna’s hummingbird	*Calypte anna*	Apodiformes	33	32,060	sequenced
red-crested turaco	*Tauraco erythrolophus*	Musophagiformes	33	32,247	–
American flamingo	*Phoenicopterus ruber*	Phoenicopteriformes	33	31,928	–
golden eagle	*Aquila chrysaetos*	Accipitriformes	28	32,031	–

^1^ NCBI, National Center for Biotechnology Information. NCBI Genome data are given according to the NCBI’s Genome resource and NCBI Datasets [55].

**Table 2:** Intrachromosomal (intra) and interchromosomal (inter) rearrangements in the genomes of eight bird species (according to [17,87]).

Species	Karyotype (2*n*)	*R*_1_, % ^1^	Inversions	Duplications	Translocations		Fusions	Fissions	Total Rearrangements		
					Intra	Inter			Intra	Inter	All
chicken	78	100	3	–	–	–	1	–	3	1	4
guineafowl	78	100	4	–	–	–	2	–	4	2	6
duck	80	85.1	8	–	–	–	–	–	8	0	8
pigeon	80	93.2	11	–	–	–	–	–	11	0	11
houbara	78	87.8	9	–	–	–	–	–	9	0	9
blackbird	80	78.4	9	–	–	–	–	1	9	1	10
canary	80	73.0	4	2	2	2	–	1	8	3	11
woodcock	96	73.0	8	–	–	3	–	5	8	8	16

^1^ *R*_1_, success rate of interspecific FISH hybridization using chicken probes.

**Table 3:** The results of screening the white-throated sparrow genomic BAC library (according to [78,150]).

Chromosomes	No. of Probes Used	No. of Successful Probes	Percentage of Successful DNA Hybridization	No. of Positive BAC Clones	Positive BAC Clones per Probe
Macrochromosomes (GGA1–GGA5)	147	46	31.29%	390	8.48
Intermediate chromosomes (GGA6–GGA10)	18	5	27.78%	40	8.00
Microchromosomes (GGA11–GGA28, GGA33)	43	20	46.51%	178	8.90
Sex chromosomes (GGAZ, GGAW)	8	6	75.00%	32	5.33

**Table 4:** Probe success rate (%) after cross-species DNA hybridization (according to [78,150]). The results are shown as a function of the probe species specificity and the efficiency of successful probes by species and by sequence type.

Cross-Species DNA Hybridization	Divergence Time, Million Years Ago ^1^	No. of Probes by Species	No. of Successful Probes	Percentage Relative to Probes by Species	Probe Sequence Type	No. of Successful Probes by Type	Percentage of Relatively Successful Probes
Chicken–sparrow	98.0	194	65	33.5%	chicken overgos		
					coding regions	47	72.3%
					5’ and 3’ UTR	6	9.2%
					introns	4	6.1%
					other non-coding regions	8	12.3%
Turkey-spa–row	98.0	19	9	47.4%	turkey overgos		
					coding regions	9	100%
Zebra finch–sparrow	38.0	3	3	100%	zebra finch overgos		
					coding regions	3	100%

^1^ The divergence estimate is taken from the TimeTree database [152,153,154].

## Data Availability

Not applicable.

## References

[1] Griffin D.K., Farré M., Lithgow P., O’Connor R., Romanov M.N., Larkin D. (2015). Avian chromonomics goes functional. Chromosome Res..

[2] Souza G.M., Vidal J.A.D., Utsunomia R., Deon G.A., de Oliveira E.H.C., Franca R.T., Porto-Foresti F., Liehr T., de Souza F.H.S., Kretschmer R. (2025). Cytogenomic analysis in Seriemas (Cariamidae): Insights into an atypical avian karyotype. J. Hered..

[3] O’Connor R.E., Kretschmer R., Romanov M.N., Griffin D.K. (2024). A bird’s-eye view of chromosomic evolution in the Class Aves. Cells.

[4] Romanov M.N., Trukhina A.V., Smirnov A.F., Griffin D.K., Pozyabin S.V., Kochish I.I., Romanov M.N. (2021). Actual Problems of Cytogenomics, Organization and Evolution of the Genomes and Chromosomes in Birds. Molecular Genetic Technologies for Analysis of Gene Expression Related to Animal Productivity and Disease Resistance; Proceedings of the 3rd International Scientific and Practical Conference, Moscow, Russia, 30 September 2021.

[5] Dodgson J.B., Romanov M.N. (2004). Use of chicken models for the analysis of human disease. Curr. Protoc. Hum. Genet..

[6] Schmid M., Smith J., Burt D.W., Aken B.L., Antin P.B., Archibald A.L., Ashwell C., Blackshear P.J., Boschiero C., Brown C.T. (2015). Third Report on Chicken Genes and Chromosomes 2015. Cytogenet. Genome Res..

[7] Warren W.C., Hillier L.W., Tomlinson C., Minx P., Kremitzki M., Graves T., Markovic C., Bouk N., Pruitt K.D., Thibaud-Nissen F. (2017). A new chicken genome assembly provides insight into avian genome structure. G3.

[8] Huang Z., Xu Z., Bai H., Huang Y., Kang N., Ding X., Liu J., Luo H., Yang C., Chen W. (2023). Evolutionary analysis of a complete chicken genome. Proc. Natl. Acad. Sci. USA.

[9] Jarvis E.D., Mirarab S., Aberer A.J., Li B., Houde P., Li C., Ho S.Y., Faircloth B.C., Nabholz B., Howard J.T. (2014). Whole-genome analyses resolve early branches in the tree of life of modern birds. Science.

[10] Prum R.O., Berv J.S., Dornburg A., Field D.J., Townsend J.P., Lemmon E.M., Lemmon A.R. (2015). A comprehensive phylogeny of birds (Aves) using targeted next-generation DNA sequencing. Nature.

[11] Stiller J., Feng S., Chowdhury A.A., Rivas-González I., Duchêne D.A., Fang Q., Deng Y., Kozlov A., Stamatakis A., Claramunt S. (2024). Complexity of avian evolution revealed by family-level genomes. Nature.

[12] International Chicken Genome Sequencing Consortium (2004). Sequence and comparative analysis of the chicken genome provide unique perspectives on vertebrate evolution. Nature.

[13] Lee M.K., Ren C.W., Yan B., Cox B., Zhang H.B., Romanov M.N., Sizemore F.G., Suchyta S.P., Peters E., Dodgson J.B. (2003). Construction and characterization of three BAC libraries for analysis of the chicken genome. Anim. Genet..

[14] Dodgson J.B., Romanov M.N. (2004). The Chicken Genome: From Maps to Sequence. Proceedings of the 8th International Symposium on Avian Endocrinology: Symposium Talk and Plenary Lecture Abstracts.

[15] Dodgson J.B., Romanov M.N., Sizemore F.G., Price J.A. (2003). Integration of Genetic and Physical Maps of the Chicken Genome. Proceedings of the Advances in Genome Biology and Technology, in Cooperation with Automation in Mapping and DNA Sequencing.

[16] Romanov M.N., Rondelli C.M., Dodgson J.B. (2004). Alignment of the Linkage Map, Physical Map, and Sequence of the Chicken Genome. Proceedings of the International Plant and Animal Genome XII Conference.

[17] Romanov M.N., Kiazim L., O’Connor R., Griffin D.K. (2020). Current Molecular Genetic and Genomic Technologies in the field of Studying the Avian Biology. 2. Basic Research. Molecular Genetic Technologies for Analysis of Gene Expression Related to Animal Productivity and Disease Resistance, Proceedings of the 2nd International Scientific and Practical Conference, Moscow, Russia, 25 December 2020.

[18] Martell H., O’Connor R., Damas J., Mandawala A., Fowler K., Joseph S., Farré M., Romanov M.N., Lithgow P.E., Larkin D.M. (2015). Assembling and Comparing Avian Genomes by Molecular Cytogenetics. Proceedings of the 2nd Bioinformatics Symposium.

[19] Sazanov A.A., Sazanova A.L., Romanov M.N., Stekol’nikova V.A., Malewski T., Korczak M., Jaszczak K., Smirnov A.F. Molecular Organization of Chicken Genome. Proceedings of the Genomic and Microarray Analysis in Biology and Medicine.

[20] Romanov M.N., Griffin D.K., Pozyabin S.V., Kochish I.I., Romanov M.N. (2021). Molecular Genetic and Genomic Approaches to Studying Evolution and Adaptation in Birds. Molecular Genetic Technologies for Analysis of Gene Expression Related to Animal Productivity and Disease Resistance, Proceedings of the 3rd International Scientific and Practical Conference, Moscow, Russia, 30 September, 2021.

[21] Gill F., Donsker D., Rasmussen P. (2025). IOC World Bird List (v15.1).

[22] Tsuda Y., Nishida-Umehara C., Ishijima J., Yamada K., Matsuda Y. (2007). Comparison of the Z and W sex chromosomal architectures in elegant crested tinamou (*Eudromia elegans*) and ostrich (*Struthio camelus*) and the process of sex chromosome differentiation in palaeognathous birds. Chromosoma.

[23] Warren W.C., Clayton D.F., Ellegren H., Arnold A.P., Hillier L.W., Künstner A., Searle S., White S., Vilella A.J., Fairley S. (2010). The genome of a songbird. Nature.

[24] Dalloul R.A., Long J.A., Zimin A.V., Aslam L., Beal K., Ann Blomberg L., Bouffard P., Burt D.W., Crasta O., Crooijmans R.P. (2010). Multi-platform next-generation sequencing of the domestic turkey (*Meleagris gallopavo*): Genome assembly and analysis. PLoS Biol..

[25] Romanov M.N., Dodgson J.B. (2005). Development of a Physical and Comparative Map of the Turkey Genome. Proceedings of the International Plant and Animal Genome XIII Conference.

[26] Romanov M.N., Dodgson J.B. (2006). Cross-species overgo hybridization and comparative physical mapping within avian genomes. Anim. Genet..

[27] Zhang G., Jarvis E.D., Gilbert M.T.P. (2014). Avian genomes. A flock of genomes. Introduction. Science.

[28] Zhang G., Li C., Li Q., Li B., Larkin D.M., Lee C., Storz J.F., Antunes A., Greenwold M.J., Meredith R.W. (2014). Comparative genomics reveals insights into avian genome evolution and adaptation. Science.

[29] Romanov M.N., Farré M., Lithgow P.E., Fowler K.E., Skinner B.M., O’Connor R., Fonseka G., Backström N., Matsuda Y., Nishida C. (2014). Reconstruction of gross avian genome structure, organization and evolution suggests that the chicken lineage most closely resembles the dinosaur avian ancestor. BMC Genom..

[30] Li B.P., Kang N., Xu Z.X., Luo H.R., Fan S.Y., Ao X.H., Li X., Han Y.P., Ou X.B., Xu L.H. (2025). Transposable elements shape the landscape of heterozygous structural variation in a bird genome. Zool. Res..

[31] Romanov M.N., Farré-Belmonte M., Lithgow P.E., O’Connor R., Fowler K.E., Larkin D.M., Griffin D.K. (2014). In silico Reconstruction of Chromosomal Rearrangements and an Avian Ancestral Karyotype. Proceedings of the International Plant and Animal Genome XXII Conference.

[32] Kadi F., Mouchiroud D., Sabeur G., Bernardi G. (1993). The compositional patterns of the avian genomes and their evolutionary implications. J. Mol. Evol..

[33] Schmidt C.J., Romanov M., Ryder O., Magrini V., Hickenbotham M., Glasscock J., McGrath S., Mardis E., Stein L.D. (2008). Gallus GBrowse: A unified genomic database for the chicken. Nucleic Acids Res..

[34] Burt D.W., White S.J. (2007). Avian genomics in the 21st century. Cytogenet. Genome Res..

[35] Wallis J.W., Aerts J., Groenen M.A., Crooijmans R.P., Layman D., Graves T.A., Scheer D.E., Kremitzki C., Fedele M.J., Mudd N.K. (2004). A physical map of the chicken genome. Nature.

[36] Habermann F.A., Cremer M., Walter J., Kreth G., von Hase J., Bauer K., Wienberg J., Cremer C., Cremer T., Solovei I. (2001). Arrangements of macro- and microchromosomes in chicken cells. Chromosome Res..

[37] Waters P.D., Patel H.R., Ruiz-Herrera A., Álvarez-González L., Lister N.C., Simakov O., Ezaz T., Kaur P., Frere C., Grützner F. (2021). Microchromosomes are building blocks of bird, reptile, and mammal chromosomes. Proc. Natl. Acad. Sci. USA.

[38] Álvarez-González L., Ruiz-Herrera A. (2025). Evolution of 3D chromatin folding. Annu. Rev. Anim. Biosci..

[39] Völker M., Backström N., Skinner B.M., Langley E.J., Bunzey S.K., Ellegren H., Griffin D.K. (2010). Copy number variation, chromosome rearrangement, and their association with recombination during avian evolution. Genome Res..

[40] Bickmore W.A., van Steensel B. (2013). Genome architecture: Domain organization of interphase chromosomes. Cell.

[41] Kapusta A., Suh A. (2017). Evolution of bird genomes—A transposon’s-eye view. Ann. N. Y. Acad. Sci..

[42] Sotero-Caio C.G., Platt R.N., Suh A., Ray D.A. (2017). Evolution and diversity of transposable elements in vertebrate genomes. Genome Biol. Evol..

[43] de Oliveira Furo I., Kretschmer R., Dos Santos M.S., de Lima Carvalho C.A., Gunski R.J., O’Brien P.C.M., Ferguson-Smith M.A., Cioffi M.B., de Oliveira E.H.C. (2017). Chromosomal mapping of repetitive DNAs in *Myiopsitta monachus* and *Amazona aestiva* (Psittaciformes, Psittacidae) with emphasis on the sex chromosomes. Cytogenet. Genome Res..

[44] Kretschmer R., de Oliveira T.D., de Oliveira Furo I., Oliveira Silva F.A., Gunski R.J., del Valle Garnero A., de Bello Cioffi M., de Oliveira E.H.C., de Freitas T.R.O. (2018). Repetitive DNAs and shrink genomes: A chromosomal analysis in nine Columbidae species (Aves, Columbiformes). Genet. Mol. Biol..

[45] Kretschmer R., Toma G.A., Deon G.A., dos Santos N., dos Santos R.Z., Utsunomia R., Porto-Foresti F., Gunski R.J., Garnero A.D.V., Liehr T. (2024). Satellitome analysis in the southern lapwing (*Vanellus chilensis*) genome: Implications for satDNA evolution in charadriiform birds. Genes.

[46] Zhou Q., Zhang J., Bachtrog D., An N., Huang Q., Jarvis E.D., Gilbert M.T.P., Zhang G. (2014). Complex evolutionary trajectories of sex chromosomes across bird taxa. Science.

[47] Peona V., Palacios-Gimenez O.M., Blommaert J., Liu J., Haryoko T., Jønsson K.A., Irestedt M., Zhou Q., Jern P., Suh A. (2021). The avian W chromosome is a refugium for endogenous retroviruses with likely effects on female-biased mutational load and genetic incompatibilities. Philos. Trans. R. Soc. B Biol. Sci..

[48] Ayers K.L., Davidson N.M., Demiyah D., Roeszler K.N., Grützner F., Sinclair A.H., Oshlack A., Smith C.A. (2013). RNA sequencing reveals sexually dimorphic gene expression before gonadal differentiation in chicken and allows comprehensive annotation of the W-chromosome. Genome Biol..

[49] Green R.E., Braun E.L., Armstrong J., Earl D., Nguyen N., Hickey G., Vandewege M.W., St. John J.A., Capella-Gutiérrez S., Castoe T.A. (2014). Three crocodilian genomes reveal ancestral patterns of evolution among archosaurs. Science.

[50] Ellegren H., Smeds L., Burri R., Olason P.I., Backström N., Kawakami T., Künstner A., Mäkinen H., Nadachowska-Brzyska K., Qvarnström A. (2012). The genomic landscape of species divergence in *Ficedula* flycatchers. Nature.

[51] Oleksyk T.K., Pombert J.F., Siu D., Mazo-Vargas A., Ramos B., Guiblet W., Afanador Y., Ruiz-Rodriguez C.T., Nickerson M.L., Logue D.M. (2012). A locally funded Puerto Rican parrot (*Amazona vittata*) genome sequencing project increases avian data and advances young researcher education. Gigascience.

[52] Rands C.M., Darling A., Fujita M., Kong L., Webster M.T., Clabaut C., Emes R.D., Heger A., Meader S., Hawkins M.B. (2013). Insights into the evolution of Darwin’s finches from comparative analysis of the *Geospiza magnirostris* genome sequence. BMC Genomics.

[53] Shapiro M.D., Kronenberg Z., Li C., Domyan E.T., Pan H., Campbell M., Tan H., Huff C.D., Hu H., Vickrey A.I. (2013). Genomic diversity and evolution of the head crest in the rock pigeon. Science.

[54] Damas J., Farré M., Lithgow P., Romanov M., Li C., Griffin D.K., Larkin D.M. (2015). Towards the construction of avian chromosome assemblies. Chromosome Res..

[55] NCBI (2025). Genomic Data Available from NCBI Datasets. Genome, National Center for Biotechnology Information, National Library of Medicine, Bethesda, MD, USA. https://www.ncbi.nlm.nih.gov/datasets/genome/.

[56] Ellegren H. (2013). The evolutionary genomics of birds. Annu. Rev. Ecol. Evol. Syst..

[57] Ganapathy G., Howard J.T., Ward J.M., Li J., Li B., Li Y., Xiong Y., Zhang Y., Zhou S., Schwartz D.C. (2014). High-coverage sequencing and annotated assemblies of the budgerigar genome. Gigascience.

[58] Zhang G., Rahbek C., Graves G.R., Lei F., Jarvis E.D., Gilbert M.T.P. (2015). Genomics: Bird sequencing project takes off. Nature.

[59] Griffin D.K., Farré M., Lithgow P., O’Connor R., Fowler K., Romanov M., Larkin D. (2014). Avian cytogenetics goes functional. Chromosome Res..

[60] Romanov M.N., Griffin D.K. (2015). The use of avian BAC libraries and clones. Cytogenet. Genome Res..

[61] Romanov M.N., Farré M., Lithgow P.E., O’Connor R., Fowler K.E., Skinner B.M., Larkin D.M., Griffin D.K. (2015). Avian ancestral karyotype reconstruction and differential rates of inter- and intrachromosomal change in different lineages. Chromosome Res..

[62] Kumar S., Hedges S.B. (1998). A molecular timescale for vertebrate evolution. Nature.

[63] Pereira S.L., Baker A.J. (2006). A mitogenomic timescale for birds detects variable phylogenetic rates of molecular evolution and refutes the standard molecular clock. Mol. Biol. Evol..

[64] Schmid M., Nanda I., Hoehn H., Schartl M., Haaf T., Buerstedde J.M., Arakawa H., Caldwell R.B., Weigend S., Burt D.W. (2005). Second Report on Chicken Genes and Chromosomes 2005. Cytogenet. Genome Res..

[65] Hedges S.B., Kumar S. (2009). The Timetree of Life.

[66] Griffin D.K., O’Connor R., Romanov M.N., Damas J., Farré M., Martell H., Kiazim L.G., Jennings R., Mandawala A.A., Joseph S. (2018). Jurassic spark: Mapping the genomes of birds and other dinosaurs. Comp. Cytogenet..

[67] O’Connor R., Romanov M.N., Farré M., Larkin D.M., Griffin D.K. (2016). Gross genome evolution in the Dinosauria. Chromosome Res..

[68] Clarke J.A., Tambussi C.P., Noriega J.I., Erickson G.M., Ketcham R.A. (2005). Definitive fossil evidence for the extant avian radiation in the Cretaceous. Nature.

[69] Torres C.R., Clarke J.A., Groenke J.R., Lamanna M.C., MacPhee R.D., Musser G.M., Roberts E.M., O’Connor P.M. (2025). Cretaceous Antarctic bird skull elucidates early avian ecological diversity. Nature.

[70] O’Connor J. (2025). Archaeopteryx. Curr. Biol..

[71] O’Connor J., Clark A., Kuo P.C., Kiat Y., Fabbri M., Shinya A., Van Beek C., Lu J., Wang M., Hu H. (2025). Chicago *Archaeopteryx* informs on the early evolution of the avian bauplan. Nature.

[72] Matsuda Y., Nishida-Umehara C., Tarui H., Kuroiwa A., Yamada K., Isobe T., Ando J., Fujiwara A., Hirao Y., Nishimura O. (2005). Highly conserved linkage homology between birds and turtles: Bird and turtle chromosomes are precise counterparts of each other. Chromosome Res..

[73] O’Connor R.E., Romanov M.N., Farré M., Larkin D.M., Griffin D.K. (2015). Reconstruction of the putative Saurian karyotype and the hypothetical chromosome rearrangements that occurred along the Dinosaur lineage. Chromosome Res..

[74] O’Connor R., Damas J., Farré M., Romanov M.N., Martell H., Fonseka G., Jennings R., Kiazam L., Bennett S., Ward J. (2016). Upgrading molecular cytogenetics to study reproduction and reproductive isolation in mammals, birds, and dinosaurs. Cytogenet. Genome Res..

[75] Modi W.S., Romanov M., Green E.D., Ryder O. (2009). Molecular cytogenetics of the California condor: Evolutionary and conservation implications. Cytogenet. Genome Res..

[76] Romanov M.N., Ryder O.A., Koriabine M., Nefedov M., de Jong P.J., Dodgson J.B. (2006). Comparative Physical Mapping in Avians and Conservation Genomics of California Condor. Proceedings of the International Plant and Animal Genome XIV Conference.

[77] Romanov M.N., Koriabine M., Nefedov M., de Jong P.J., Ryder O.A. (2006). Construction of a California condor BAC library and first-generation chicken–condor comparative physical map as an endangered species conservation genomics resource. Genomics.

[78] Romanov M.N., Dodgson J.B., Gonser R.A., Tuttle E.M. (2011). Comparative BAC-based mapping in the white-throated sparrow, a novel behavioral genomics model, using interspecies overgo hybridization. BMC Res. Notes.

[79] Lithgow P.E., O’Connor R., Smith D., Fonseka G., Al Mutery A., Rathje C., Frodsham R., O’Brien P., Kasai F., Ferguson-Smith M.A. (2014). Novel tools for characterising inter and intra chromosomal rearrangements in avian microchromosomes. Chromosome Res..

[80] O’Connor R.E., Farré M., Joseph S., Damas J., Kiazim L., Jennings R., Bennett S., Slack E.A., Allanson E., Larkin D.M. (2018). Chromosome-level assembly reveals extensive rearrangement in saker falcon and budgerigar, but not ostrich, genomes. Genome Biol..

[81] O’Connor R.E., Kiazim L., Skinner B., Fonseka G., Joseph S., Jennings R., Larkin D.M., Griffin D.K. (2019). Patterns of microchromosome organization remain highly conserved throughout avian evolution. Chromosoma.

[82] Kretschmer R., de Souza M.S., Furo I.d.O., Romanov M.N., Gunski R.J., Garnero A.d.V., de Freitas T.R.O., de Oliveira E.H.C., O’Connor R.E., Griffin D.K. (2021). Interspecies chromosome mapping in Caprimulgiformes, Piciformes, Suliformes, and Trogoniformes (Aves): Cytogenomic insight into microchromosome organization and karyotype evolution in birds. Cells.

[83] Blagoveshchenskiĭ I.I., Sazanova A.L., Romanov M.N., Fomichev K.A., Stekol’nikova V.A., Sazanov A.A. (2008). Cytogenetic Localization of the Genes on Avian Z and W Chromosomes with the Use of Large Insert Genomic Clones. Problems of Biology, Ecology, Geography, Education: History and Contemporaneity, Proceedings of the 2nd International Scientific and Practical Conference, St. Petersburg, Russia, 3–5 June 2008.

[84] Blagoveshchenskiĭ I.I., Sazanova A.L., Stekol’nikova V.A., Fomichev K.A., Barkova O.I., Romanov M.N., Sazanov A.A. (2011). Investigation of Pseudoautosomal and Bordering regions in Avian Z and W Chromosomes with the Use of Large Insert Genomic BAC Clones. Genetika.

[85] Sazanov A.A., Romanov M.N., Sazanova A.L., Tzareva V.A., Kozyreva A.A., Price J.A., Smirnov A.F., Dodgson J.B. (2004). Chromosomal Localization of Continuous Genomic Clones in the Chicken with a View of Comparative Mapping. Genetics in the XXI Century: Current State and Prospects for Development, Proceedings of the III Congress of the Vavilov Society of Geneticists and Selectionists, Moscow, Russia, 6–12 June 2004.

[86] Sazanov A.A., Romanov M.N., Smirnov A.F. (2005). Libraries of large-insert genomic clones as a tool for molecular cytogenetic analysis of avian genome. Russ. J. Genet..

[87] Kiazim L.G., O’Connor R.E., Larkin D.M., Romanov M.N., Narushin V.G., Brazhnik E.A., Griffin D.K. (2021). Comparative mapping of the macrochromosomes of eight avian species provides further insight into their phylogenetic relationships and avian karyotype evolution. Cells.

[88] Sazanova A.L., Romanov M.N., Blagoveshenski I.Y., Fomichev K.A., Stekol’nikova V.A., Nefedov M., de Jong P.J., Modi W.S., Ryder O.A., Dodgson J.B. (2008). Cytogenetic Localization of avian Z- and W-linked Genes Using Large Insert BAC Clones. Proceedings of the International Plant and Animal Genome XVI Conference.

[89] Lithgow P.E., O’Connor R., Smith D., Fonseka G., Rathje C., Frodsham R., O’Brien P.C., Ferguson-Smith M.A., Skinner B.M., Griffin D.K. (2014). Novel Tools for Characterising Inter- and Intra-chromosomal Rearrangements in Avian Microchromosomes. Proceedings of the 2014 Meeting on Avian Model Systems.

[90] Alves Barcellos S., Kretschmer R., Santos de Souza M., Tura V., Pozzobon L.C., Ochotorena de Freitas T.R., Griffin D.K., O’Connor R., Gunski R.J., del Valle Garnero A. (2024). Understanding microchromosomal organization and evolution in four representative woodpeckers (Picidae, Piciformes) through BAC-FISH analysis. Genome.

[91] Adolfsson S., Ellegren H. (2013). Lack of dosage compensation accompanies the arrested stage of sex chromosome evolution in ostriches. Mol. Biol. Evol..

[92] Nishida-Umehara C., Tsuda Y., Ishijima J., Ando J., Fujiwara A., Matsuda Y., Griffin D.K. (2007). The molecular basis of chromosome orthologies and sex chromosomal differentiation in palaeognathous birds. Chromosome Res..

[93] Hansmann T., Nanda I., Volobouev V., Yang F., Schartl M., Haaf T., Schmid M. (2009). Cross-species chromosome painting corroborates microchromosome fusion during karyotype evolution of birds. Cytogenet. Genome Res..

[94] Nishida C., Ishijima J., Kosaka A., Tanabe H., Habermann F.A., Griffin D.K., Matsuda Y. (2008). Characterization of chromosome structures of Falconinae (Falconidae, Falconiformes, Aves) by chromosome painting and delineation of chromosome rearrangements during their differentiation. Chromosome Res..

[95] Joseph S., O’Connor R.E., Al Mutery A.F., Watson M., Larkin D.M., Griffin D.K. (2018). Chromosome level genome assembly and comparative genomics between three falcon species reveals an unusual pattern of genome organisation. Diversity.

[96] Nie W., O’Brien P.C., Ng B.L., Fu B., Volobouev V., Carter N.P., Ferguson-Smith M.A., Yang F. (2009). Avian comparative genomics: Reciprocal chromosome painting between domestic chicken (*Gallus gallus*) and the stone curlew (*Burhinus oedicnemus*, Charadriiformes)—An atypical species with low diploid number. Chromosome Res..

[97] Kretschmer R., Souza M.S.D., Barcellos S.A., Degrandi T.M., Pereira J.C., O’Brien P., Ferguson-Smith M.A., Gunski R.J., Garnero A.D.V., Oliveira E.H.C.D. (2020). Novel insights into chromosome evolution of Charadriiformes: Extensive genomic reshuffling in the wattled jacana (*Jacana jacana*, Charadriiformes, Jacanidae). Gen. Mol. Biol..

[98] Duchêne D.A., Chowdhury A.A., Yang J., Iglesias-Carrasco M., Stiller J., Feng S., Bhatt S., Gilbert M.T.P., Zhang G., Tobias J.A. (2025). Drivers of avian genomic change revealed by evolutionary rate decomposition. Nature.

[99] Torgasheva A.A., Malinovskaya L.P., Zadesenets K.S., Karamysheva T.V., Kizilova E.A., Akberdina E.A., Pristyazhnyuk I.E., Shnaider E.P., Volodkina V.A., Saifitdinova A.F. (2019). Germline-restricted chromosome (GRC) is widespread among songbirds. Proc. Natl. Acad. Sci. USA.

[100] Kinsella C.M., Ruiz-Ruano F.J., Dion-Côté A.M., Charles A.J., Gossmann T.I., Cabrero J., Kappei D., Hemmings N., Simons M.J., Camacho J.P.M. (2019). Programmed DNA elimination of germline development genes in songbirds. Nat. Commun..

[101] Sotelo-Muñoz M., Poignet M., Albrecht T., Kauzál O., Dedukh D., Schlebusch S.A., Janko K., Reifová R. (2022). Germline-restricted chromosome shows remarkable variation in size among closely related passerine species. Chromosoma.

[102] Schlebusch S.A., Rídl J., Poignet M., Ruiz-Ruano F.J., Reif J., Pajer P., Pačes J., Albrecht T., Suh A., Reifová R. (2023). Rapid gene content turnover on the germline-restricted chromosome in songbirds. Nat. Commun..

[103] Bravo G.A., Schmitt C.J., Edwards S.V. (2021). What have we learned from the first 500 avian genomes?. Annu. Rev. Ecol. Evol. Syst..

[104] Borodin P., Chen A., Forstmeier W., Fouché S., Malinovskaya L., Pei Y., Reifová R., Ruiz-Ruano F.J., Schlebusch S.A., Sotelo-Muñoz M. (2022). Mendelian nightmares: The germline-restricted chromosome of songbirds. Chromosome Res..

[105] Seoighe C., Federspiel N., Jones T., Hansen N., Bivolarovic V., Surzycki R., Tamse R., Komp C., Huizar L., Davis R.W. (2000). Prevalence of small inversions in yeast gene order evolution. Proc. Natl. Acad. Sci. USA.

[106] Fischer G., Neuvéglise C., Durrens P., Gaillardin C., Dujon B. (2001). Evolution of gene order in the genomes of two related yeast species. Genome Res..

[107] Britten R.J., Rowen L., Williams J., Cameron R.A. (2003). Majority of divergence between closely related DNA samples is due to indels. Proc. Natl. Acad. Sci. USA.

[108] Biemont C., Vieira C. (2006). Junk DNA as an evolutionary force. Nature.

[109] Eichler E.E., Sankoff D. (2003). Structural dynamics of eukaryotic chromosome evolution. Science.

[110] Murphy W.J., Larkin D.M., der Wind A.E.V., Bourque G., Tesler G., Auvil L., Beever J.E., Chowdhary B.P., Galibert F., Gatzke L. (2005). Dynamics of mammalian chromosome evolution inferred from multispecies comparative maps. Science.

[111] Carbone L., Vessere G.M., Hallers B.F.T., Zhu B., Osoegawa K., Mootnick A., Kofler A., Wienberg J., Rogers J., Humphray S. (2006). A high-resolution map of synteny disruptions in gibbon and human genomes. PLoS Genet..

[112] Carbone L., Nergadze S.G., Magnani E., Misceo D., Cardone M.F., Roberto R., Bertoni L., Attolini C., Piras M.F., de Jong P. (2006). Evolutionary movement of centromeres in horse, donkey, and zebra. Genomics.

[113] Cardone M.F., Alonso A., Pazienza M., Ventura M., Montemurro G., Carbone L., de Jong P.J., Stanyon R., D’Addabbo P., Archidiacono N. (2006). Independent centromere formation in a capricious, gene-free domain of chromosome 13q21 in Old World monkeys and pigs. Genome Biol..

[114] Misceo D., Cardone M.F., Carbone L., D’Addabbo P., De Jong P.J., Rocchi M., Archidiacono N. (2005). Evolutionary history of chromosome 20. Mol. Biol. Evol..

[115] Ventura M., Weigl S., Carbone L., Cardone M.F., Misceo D., Teti M., D’Addabbo P., Wandall A., Björck E., de Jong P.J. (2004). Recurrent sites for new centromere seeding. Genome Res..

[116] Kasai F., Garcia C., Arruga M.V., Ferguson-Smith M.A. (2003). Chromosome homology between chicken (*Gallus gallus domesticus*) and the red-legged partridge (*Alectoris rufa*); evidence of the occurrence of a neocentromere during evolution. Cytogenet. Genome Res..

[117] Sazanov A.A., Sazanova A.L., Tzareva V.A., Kozyreva A.A., Smirnov A.F., Romanov M.N., Price J.A., Dodgson J.B. (2004). Chromosomal Localization of Large Insert Clones of the Chicken Genome: Expanding the Comparative Map. Proceedings of the International Plant and Animal Genome XII Conference.

[118] Matzke M.A., Varga F., Berger H., Schernthaner J., Schweizer D., Mayr B., Matzke A.J.M. (1990). A 41–42-bp tandemly repeated sequence isolated from nuclear envelopes of chicken erythrocytes is located predominantly on microchromosomes. Chromosoma.

[119] Wang Z., Miyake T., Edwards S.V., Amemiya C.T. (2006). Tuatara (*Sphenodon*) genomics: BAC library construction, sequence survey, and application to the DMRT gene family. J. Hered..

[120] Williamson S.H., Hubisz M.J., Clark A.G., Payseur B.A., Bustamante C.D., Nielsen R. (2007). Localizing recent adaptive evolution in the human genome. PLoS Genet..

[121] Galkina S., Deryusheva S., Fillon V., Vignal A., Crooijmans R., Groenen M., Rodionov A., Gaginskaya E. (2006). FISH on avian lampbrush chromosomes produces higher resolution gene mapping. Genetica.

[122] Krasikova A., Deryusheva S., Galkina S., Kurganova A., Evteev A., Gaginskaya E. (2006). On the positions of centromeres in chicken lampbrush chromosomes. Chromosome Res..

[123] Crombach A., Hogeweg P. (2007). Chromosome rearrangements and the evolution of genome structuring and adaptability. Mol. Biol. Evol..

[124] Kazazian H.H. (2004). Mobile elements: Drivers of genome evolution. Science.

[125] Dodgson J.B., Romanov M.N., Rondelli C.M. (2004). Integration of Chicken Linkage and Physical Maps and Sequence Alignment Using Overgo Hybridization. Proceedings of the International Plant and Animal Genome XII Conference.

[126] Farré M., Narayan J., Slavov G.T., Damas J., Auvil L., Li C., Jarvis E.D., Burt D.W., Griffin D.K., Larkin D.M. (2016). Novel insights into chromosome evolution in birds, archosaurs, and reptiles. Genome Biol. Evol..

[127] Damas J., Kim J., Farré M., Griffin D.K., Larkin D.M. (2018). Reconstruction of avian ancestral karyotypes reveals differences in the evolutionary history of macro- and microchromosomes. Genome Biol..

[128] Larkin D.M., Pape G., Donthu R., Auvil L., Welge M., Lewin H.A. (2009). Breakpoint regions and homologous synteny blocks in chromosomes have different evolutionary histories. Genome Res..

[129] Dixon J.R., Selvaraj S., Yue F., Kim A., Li Y., Shen Y., Hu M., Liu J.S., Ren B. (2012). Topological domains in mammalian genomes identified by analysis of chromatin interactions. Nature.

[130] Franke M., Ibrahim D.M., Andrey G., Schwarzer W., Heinrich V., Schöpflin R., Kraft K., Kempfer R., Jerković I., Chan W.L. (2016). Formation of new chromatin domains determines pathogenicity of genomic duplications. Nature.

[131] Delbridge M.L., Patel H.R., Waters P.D., McMillan D.A., Graves J.A.M. (2009). Does the human X contain a third evolutionary block? Origin of genes on human Xp11 and Xq28. Genome Res..

[132] Modi W.S., Crews D. (2005). Sex chromosomes and sex determination in reptiles: Commentary. Curr. Opin. Genet. Dev..

[133] Trukhina A., Smirnov A. (2014). Problems of birds sex determination. Nat. Sci..

[134] Nakagawa S. (2004). Is avian sex determination unique?: Clues from a warbler and from chickens. Trends Genet..

[135] Vicoso B., Kaiser V.B., Bachtrog D. (2013). Sex-biased gene expression at homomorphic sex chromosomes in emus and its implication for sex chromosome evolution. Proc. Natl. Acad. Sci. USA.

[136] Guioli S., Lovell-Badge R., Turner J.M. (2012). Error-prone ZW pairing and no evidence for meiotic sex chromosome inactivation in the chicken germ line. PLoS Genet..

[137] Küpper C., Augustin J., Edwards S., Székely T., Kosztolányi A., Burke T., Janes D.E. (2012). Triploid plover female provides support for a role of the W chromosome in avian sex determination. Biol. Lett..

[138] Reed K.J., Sinclair A.H. (2002). FET-1: A novel W-linked, female specific gene up-regulated in the embryonic chicken ovary. Mech. Dev..

[139] Yamada K., Nishida-Umehara C., Ishijima J., Murakami T., Shibusawa M., Tsuchiya K., Tsudzuki M., Matsuda Y. (2006). A novel family of repetitive DNA sequences amplified site-specifically on the W chromosomes in Neognathous birds. Chromosome Res..

[140] Nanda I., Schlegelmilch K., Haaf T., Schartl M., Schmid M. (2008). Synteny conservation of the Z chromosome in 14 avian species (11 families) supports a role for Z dosage in avian sex determination. Cytogenet. Genome Res..

[141] Rutkowska J., Lagisz M., Nakagawa S. (2012). The long and the short of avian W chromosomes: No evidence for gradual W shortening. Biol. Lett..

[142] Gunski R.J., Delgado Cañedo A., Del Valle Garnero A., Ledesma M.A., Coria N., Montalti D., Degrandi T.M. (2017). Multiple sex chromosome system in penguins (*Pygoscelis*, Spheniscidae). Comp. Cytogenet..

[143] Pozzobon L.C., Toma G.A., Cioffi M.D.B., de Oliveira E.H.C., Kretschmer R., de Freitas T.R.O. (2025). Karyotype evolution of Suliformes and description of a ♂Z_1_Z_1_Z_2_Z_2_/♀Z_1_Z_2_W multiple sex chromosome system in boobies (*Sula* spp.). Genome.

[144] Smeds L., Warmuth V., Bolivar P., Uebbing S., Burri R., Suh A., Nater A., Bureš S., Garamszegi L.Z., Hogner S. (2015). Evolutionary analysis of the female-specific avian W chromosome. Nat. Commun..

[145] Xu L., Auer G., Peona V., Suh A., Deng Y., Feng S., Zhang G., Blom M.P., Christidis L., Prost S. (2019). Dynamic evolutionary history and gene content of sex chromosomes across diverse songbirds. Nat. Ecol. Evol..

[146] Huerta-Cepas J., Serra F., Bork P. (2016). ETE 3: Reconstruction, analysis, and visualization of phylogenomic data. Mol. Biol. Evol..

[147] Damas J., O’Connor R., Farré M., Lenis V.P.E., Martell H.J., Mandawala A., Fowler K., Joseph S., Swain M.T., Griffin D.K. (2017). Upgrading short-read animal genome assemblies to chromosome level using comparative genomics and a universal probe set. Genome Res..

[148] Hu F., Lin Y., Tang J. (2014). MLGO: Phylogeny reconstruction and ancestral inference from gene-order data. BMC Bioinform..

[149] Tesler G. (2002). GRIMM: Genome rearrangements web server. Bioinformatics.

[150] Romanov M.N., Narushin V.G., Gonser R.A., Tuttle E.M. (2020). [Mathematical assessment of BAC-based interspecies hybridization data in the process of genomic mapping in the white-throated sparrow as an avian behavioral model]. Molecular Genetic Technologies for Analysis of Gene Expression Related to Animal Productivity and Disease Resistance, Proceedings of the 2nd International Scientific and Practical Conference, Moscow, Russia, 25 December 2020.

[151] Wikipedia Contributors (2025). Genomic Evolution of Birds. Wikipedia, The Free Encyclopedia. https://en.wikipedia.org/w/index.php?title=Genomic_evolution_of_birds&oldid=1301065240.

[152] Kumar S., Stecher G., Suleski M., Hedges S.B. (2017). TimeTree: A resource for timelines, timetrees, and divergence times. Mol. Biol. Evol..

[153] Kumar S., Suleski M., Craig J.M., Kasprowicz A.E., Sanderford M., Li M., Stecher G., Hedges S.B. (2022). TimeTree 5: An expanded resource for species divergence times. Mol. Biol. Evol..

[154] TimeTree Timescale of Life. Institute for Genomics and Evolutionary Medicine, Center of Biodiversity, Temple University, Philadelphia, PA, USA, 2005–2025. https://timetree.org/.

[155] Seddon P.J., Seddon R.J. (1991). Chromosome analysis and sex identification of Yelloweyed Penguins *Megadyptes antipodes*. Mar. Ornithol..

[156] Romanov M.N., O’Connor R., Skinner B.M., Martell H., Farré M., Larkin D.M., Griffin D.K. (2015). Comparative Cytogenomics Enhanced with Bioinformatic Tools Provides Further Insights into Genome Evolution of Birds and Other Amniotes. Proceedings of the 2nd Annual Food, Nutrition and Agriculture Genomics Congress: Congress Workbook.

[157] Romanov M.N., Daniels L.M., Dodgson J.B., Delany M.E. (2005). Integration of the cytogenetic and physical maps of chicken chromosome 17. Chromosome Res..

